# Experimental Study on Biaxial Dynamic Compressive Properties of ECC

**DOI:** 10.3390/ma14051257

**Published:** 2021-03-06

**Authors:** Shuling Gao, Guanhua Hu

**Affiliations:** 1 Institude of Civil Engineering and Transportation, Hebei University of Technology, Tianjin 300401, China; gaoshuling@hebet.edu.cn; 2 Civil Engineering Technology Research Center of Hebei Province, Tianjin 300134, China

**Keywords:** ECC, strain rate, lateral pressure level, stress-strain curve, constitutive relationship, compressive toughness index

## Abstract

An improved hydraulic servo structure testing machine has been used to conduct biaxial dynamic compression tests on eight types of engineered cementitious composites (ECC) with lateral pressure levels of 0, 0.125, 0.25, 0.5, 0.7, 0.8, 0.9, 1.0 (the ratio of the compressive strength applied laterally to the static compressive strength of the specimen), and three strain rates of 10^−4^, 10^−3^ and 10^−2^ s^−1^. The failure mode, peak stress, peak strain, deformation modulus, stress-strain curve, and compressive toughness index of ECC under biaxial dynamic compressive stress state are obtained. The test results show that the lateral pressure affects the direction of ECC cracking, while the strain rate has little effect on the failure morphology of ECC. The growth of lateral pressure level and strain rate upgrades the limit failure strength and peak strain of ECC, and the small improvement is achieved in elastic modulus. A two-stage ECC biaxial failure strength standard was established, and the influence of the lateral pressure level and peak strain was quantitatively evaluated through the fitting curve of the peak stress, peak strain, and deformation modulus of ECC under various strain rates and lateral pressure levels. ECC’s compressive stress-strain curve can be divided into four stages, and a normalized biaxial dynamic ECC constitutive relationship is established. The toughness index of ECC can be increased with the increase of lateral pressure level, while the increase of strain rate can reduce the toughness index of ECC. Under the effect of biaxial dynamic load, the ultimate strength of ECC is increased higher than that of plain concrete.

## 1. Introduction

Since the advent of engineered cementitious composites (ECC), scholars from various countries have carried out many studies on their basic mechanical properties, durability, structural applications, etc. They have achieved remarkable research results [1,2,3,4]. However, the research on ECC’s mechanical properties under multi-axis and the influence of strain rate on the mechanical properties of ECC are still insufficient. The ECC structure will not only be subjected to uniaxial static loads during service but will also be under various complex stress states, some typical examples are the Yokohama Building (Yokohama, Japan), which is added functions of increasing structural energy consumption and enhancing seismic performance [3], the bridge deck used to improve the fatigue resistance of bridges [5,6], the outer layer of the lining system used in the Hida highway tunnel (Gifu, Japan) [7], and so on. Simultaneously, in the course of their service, these building structures are often inevitably subjected to dynamic loads, such as earthquakes, vehicle impacts, and applications in the design of nuclear power plant containment and defense protection structures to resist explosion effects [8]. The uniaxial mechanical properties of ECC materials are quite different from those of ordinary concrete. Therefore, it is impossible to directly analyze the ECC structure by applying the mechanical model of concrete. It is necessary to study the mechanical properties of ECC materials under complex stress.

At present, specific research progress has been made on the multiaxial dynamic mechanical properties of concrete. Studies have shown that the peak stress and peak strain of concrete will increase with strain rate and lateral pressure. As early as 1974, Takeda et al. conducted dynamic triaxial experiments on concrete prisms. The study showed that peak stress and peak strain are affected by confining pressure and axial strain rate. The damage envelope surface has been dramatically improved, especially under compression loads with high strain rates [9]. Yamaguchi et al. also obtained similar conclusions [10]. Lu studied the biaxial compression and biaxial tension-compression properties of concrete under constant lateral pressure at different loading rates. The study denoted that with lateral pressure and loading rate growing, the average compressive strength of concrete grows. Simultaneously, the influence of lateral pressure on the peak strain is more significant than the effect of strain rate on the peak strain, and a failure criterion of concrete compressive strength considering the strain rate and lateral pressure is established [11]. On this basis, Yan studied the dynamic compressive properties of concrete under triaxial conditions and found that the dynamic strength under triaxial conditions did not increase significantly [12]. Liang [13], Guan [14], Meng [15], and others have conducted researches on biaxial compression of concrete under constant lateral pressure under different loading rates. In general, the conclusions obtained are relatively unified. Lateral compression and strain rate positively affect the peak stress, peak strain, and elastic modulus of concrete.

Some scholars have studied the mechanical properties of ECC under biaxial conditions. Raymond studied the mechanical behavior of HPFRCC slabs under biaxial conditions and found that HPFRCC has better biaxial compression performance than ordinary concrete, and established a biaxial failure curve [16]. Pan also got a similar conclusion [17]. Li studied ECC’s behavior under triaxial compression under equivalent confining pressure and found that with the increase of confining pressure, the ultimate strength and peak strain of ECC increased significantly [18]. Kittnun found that short fibers can dramatically improve the strength and ductility under uniaxial and biaxial loading paths [19]. Simultaneously, regarding the effect of loading rate on the tensile properties of ECC, Alessio found that the crack growth morphology of ECC is significantly different at high and low strain rates. At high strain rates, the peak intensity increases significantly [20]. Regarding the effect of loading rate on ECC compression performance, Yang established the functional relationship between the dynamic strength growth factor (DIF) and dynamic strain growth factor (DEIF) of PVA-FRCC and HFRCC materials and evaluated the dynamic compressive toughness of PVA-FRCC and HFRCC [21]. Zhao analyzed the influence of different fiber content on the dynamic mechanical properties of UHTCC, and the results showed that the dynamic compressive toughening effect of steel fiber is more significant [22]. Xu studied the effects of fiber content, humidity, and strain rate on the dynamic compressive strength, peak strain, and elastic modulus of ECC, and found that ECC is a strain-rate sensitive material [23]. The dynamic compression mechanical properties of ECC are numerically simulated, and the crack propagation and compression failure process of the specimen was simulated by Xu [24]. However, these studies currently only explore the effects of biaxial or different strain rates on ECC’s mechanical properties. The study of mechanical properties under the combined action of lateral pressure and strain rate is still at a blank stage. ECC is often subjected to various complex multiaxial stress environments in practical applications and may be affected by multiple strain rate loads. ECC is a rate-sensitive material. In fact, the strain rate encountered by the structure varies greatly. For example, the creep strain rate is lower than 10^−6^ s^−1^, and the strain rate under impact load reaches more than 10^2^ s^−1^. The strain rate under seismic load is about 10^−3^ s^−1^~10^−2^ s^−1^. The strength, stiffness, and ductility of ECC structure will be affected by strain rate. The text focuses on studying the dynamic strength characteristics and failure characteristics of ECC under constant lateral pressure within the strain rate range (10^−4^~10^−2^ s^−1^) considered by the seismic load. Therefore, this paper conducts experimental research on the biaxial dynamic mechanical properties of ECC.

This paper uses the structural testing machine reconstructed by Hebei University of Technology to carry out experimental research on ECC’s biaxial dynamic pressure resistance. A series of dynamic compression tests (strain rate of 10^−4^, 10^−3^, 10^−2^ s^−1^) under constant lateral pressure (C = *σ*_2_/*f*_c_ = 0, 0.125, 0.25, 0.5, 0.7, 0.8, 0.9, 1.0) were carried out using 100 × 100 × 100 mm^3^ ECC cube specimens, and eight lateral pressure levels were considered. Via experiments and changes of the ECC specimens, the mechanical parameters are represented, such as the failure form, compressive strength, elastic modulus, peak strain, stress-strain curve, and other ECC specimens under biaxial dynamics under different constant lateral pressures. Based on the test results, the ECC biaxial compressive strength failure criterion was proposed. The biaxial dynamic strength of ECC was evaluated by the dynamic strength growth factor *DIF* − *σ*, the biaxial dynamic peak strain of ECC was considered by the dynamic strain growth factor *DIF* − *ε*, the dynamic compression constitutive relationship of ECC was established, and the toughness index was evaluated. The influence of the lateral pressure level and strain rate on ECC toughness is discussed in this paper. In addition, we compare the difference of ultimate strength between ECC and concrete under biaxial dynamic load. This paper provides necessary experimental data for the mechanical properties of ECC under biaxial dynamics.

## 2. Materials and Methods

### 2.1. Test Raw Materials and Mix Ratio

ECC uses ordinary Portland cement (P·O 42.5°), fly ash (I grade), silica sand (70–120 mesh), silica fume with SiO_2_ content up to 95%, polycarboxylate superplasticizer, and K-II REC15 polyvinyl alcohol (PVA) fiber. Table 1 lists the material properties of PVA fibers. Table 2 shows the mixing ratio of ECC.

### 2.2. Test Device

**Figure 1:**
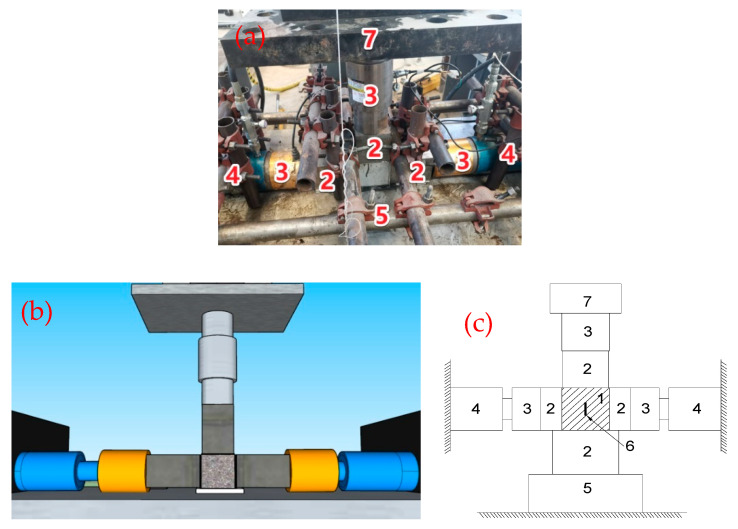
Schematic diagram of loading. (**a**) Experimental setup diagram. (**b**) 3D schematic (**c**) Plan schematic. Note: 1—engineered cementitious composite (ECC) cubic specimen; 2—Force transmission steel parts; 3—Loading sensor; 4—screw jack; 5—Lower support; 6—Foil strain gauge; 7—Vertical actuator head.

The dynamic compression test was carried out by transforming the structural testing machine in the Structural Laboratory of Hebei University of Technology (Tianjin, China). The longitudinal compression load was provided by the structural testing machine, and the lateral pressure load was realized by installing a set of lateral pressure devices. The maximum longitudinal pressure is 1000 kN, and the maximum lateral pressure is 600 kN. As shown in Figure 1, a screw jack (4) installed on the side loading frame is used to apply constant horizontal pressure. A load sensor (3) is placed between the loading plate (2) and the screw jack and connected to a voltmeter to monitor the pressure. The stress is collected by a load sensor (3) and a lateral load sensor (3) connected above the test piece, and the size of the oil valve of the jack (4) is used to control the size of the lateral pressure.

The displacement sensor built into the structural testing machine is used to measure the longitudinal displacement change, and the displacement is used as the average strain of the specimen. At the same time, a strain gauge (6) is attached to the middle of the specimen (see Figure 1) to measure the true local strain of the specimen in the middle. Data are collected by Donghua Dynamic Data Acquisition System (Version: DHDAS 6.8.21.1). Three layers of plastic film are added on each contact surface of test piece (1) and loading plate (2), and glycerin is added between each layer of plastic film to reduce friction and eliminate the influence of friction constraints on the experimental results to the greatest extent [17].

### 2.3. Test Piece Preparation and Test Method

Taking into account the equipment conditions of this experiment and the research methods of other researchers in this type of experiment, this experiment follows the current sample size 100 mm cube of the biaxial dynamic test performed by other researchers. Therefore, the size of the standard concrete cube test block under normal circumstances is not considered in this experiment, and the influence of size effect is not considered in this study. Before pouring the test piece, the ECC cube plastic mold is cleaned and brushed with a layer of mold release oil on its inner surface. The first step is to weigh the cement, fly ash, silica fume, fine sand, and then pour it into the mixer, dry the mix for 3 min, following with mixing the water reducing agent and water thoroughly and pouring it into the mixture and stir for about 5 min. Then, the final step is to add PVA fibers to stir for 8–10 min until the PVA fibers are evenly distributed. After the mixing is completed, the test pieces are poured in a 100 × 100 × 100 mm^3^ cube trial mold, and then layered. Additionally, it is still to be vibrated on the vibrating table for about 5 min until no bubbles ooze out on the surface, and smooth the surface. The ECC specimens were placed in room temperature for curing for 1–2 days, then we removed the mold number and placed it in a curing room with a temperature of 20 ± 5 °C with humidity close to 100% for 28 days. The experiment was carried out after 180 days of natural placement. Due to the limitation of experimental conditions, the strength of ECC in this experiment only represents the experimental results after 180 days, and the results may be higher than those immediately after 28-day standard curing. Before the test, the uneven surface of the specimen was polished to be completely flat, and the flatness of the ECC specimen was controlled to meet the experimental requirements. After that, all specimens were loaded on the modified structural testing machine, and the loading method was displacement control. The corresponding constant displacement rates vertically are 0.01 mm/s, 0.1 mm/s, and 1 mm/s, respectively. During the test, glycerin and plastic film were painted on all four sides of the test piece. After installation of the test piece, a load along the axial direction was applied at a fixed loading rate until the test piece was broken.

Samples were marked in C0.5T4-x format. C represents the level of lateral pressure applied to the sample; that is, the ratio of the static pressure applied laterally to the static compressive strength of the sample (C = *σ*_2_/*f*_c_). fc is the average compressive strength of the ECC cube specimens with a side length of 100 mm measured under uniaxial (C = 0). *σ*_1_ refers to the stress in the vertical direction of the specimen, and σ_2_ refers to the stress in the direction where the specimen is laterally restrained. The number after C indicates the group corresponding to the lateral pressure level. T represents the magnitude of the strain rate, and the number after T takes the absolute value after the logarithm of the strain rate to indicate the grouping of strain rates. The constant lateral pressure levels of the test are C = 0, 0.125, 0.25, 0.5, 0.7, 0.8, 0.9, 1.0 and the strain rates are 10^−4^, 10^−3^, 10^−2^ s^−1^. Please refer to Table 3 for specific loading conditions.

## 3. Results and Discussion

The critical characteristic parameters usually used to study and measure the stress-strain curve under ECC compression include peak stress, peak strain, deformation modulus, stress-strain curve, compressive toughness, etc. This chapter analyzes the experimental results of the biaxial dynamic compression test of ECC materials, explores the failure mode of the specimen, establishes the ECC biaxial failure criterion, and discusses the characteristic parameters of the specimen biaxial dynamic compression, including the peak stress of the specimen, peak strain, deformation modulus, compressive toughness, etc.

### 3.1. Damage Form

It was found that the failure surfaces of the specimen were very rough, and the PVA fibers played an essential role in the process of the destruction of the ECC specimens. Due to a large number of test pieces, this article selects some typical representative test pieces for the explanation.

**Figure 2:**
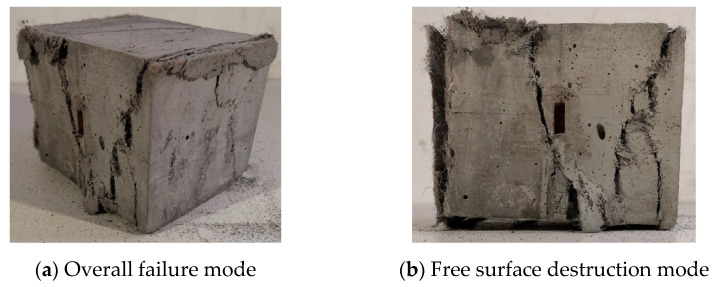
Typical uniaxial failure mode (C0T4-1) (lateral pressure level C = 0, strain rate $\dot{\varepsilon }$ = 10^−4^ s^−1^).

**Figure 3:**
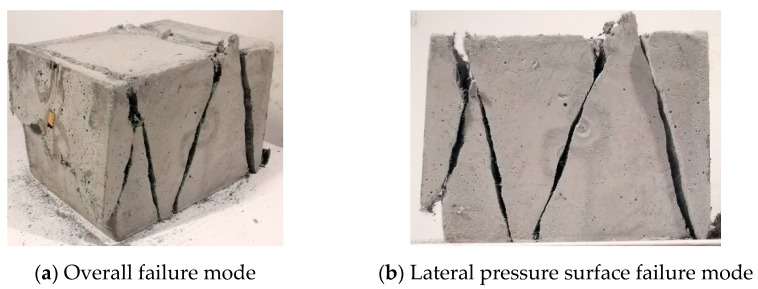
Typical biaxial failure mode (C0.8T4-8) (lateral pressure level C = 0.8, strain rate $\dot{\varepsilon }$ = 10^−4^ s^−1^).

Since the two side surfaces of the test piece are subjected to lateral force, the two surfaces subject to lateral pressure are referred to as side pressure surfaces in the following, and the two surfaces that are not in contact with the void during loading are called free surface. Figure 2 and Figure 3 show the final failure form of the ECC cube under different lateral pressure levels and different strain rates. For uniaxial compression specimens, the specimens reveal a certain degree of ductility. When the compressive strength of the specimens is reached, the specimen cracks suddenly develop and break down instantly, as shown in Figure 2. The specimen under uniaxial is mainly exhibited by shear failure, and the maximum crack penetration surface is located on the front. On the failure surface of the specimen, the PVA fiber can be clearly pulled out and broken. The main tensile stress of the specimen appears in the direction parallel to the main compressive stress σ_1_, and the principal tensile stress in the two directions can be regarded as approximately equal.

There is a clear difference between the failure modes under uniaxial and biaxial conditions, and the form of restraint has a significant influence on the failure mode. Under uniaxial, the main cracks of the specimens that eventually fail are all produced along the free surface, but in the biaxial case, as shown in Figure 3, the main cracks of the specimens only exist in the direction of the lateral pressure surface, which is due to the lateral pressure. The constraint of compressive stress *σ*_2_ produces the Poisson effect. The main tensile stress of the specimen appears on the acting surface perpendicular to *σ*_1_-*σ*_2_. Therefore, the cracking directions of the main tensile stress cracks are significantly different. When the specimen reaches the maximum bearing capacity, oblique cracks appear along the side of the specimen and which gradually penetrate and the specimen fails, and basically maintains the integrity on the free surface of the specimen, and there is basically no development of penetrating cracks on the free surface. In general, the size of the loading rate and the level of lateral pressure have no obvious effect on the final cracking failure mode of the specimen. The angle between the main tensile cracks of most specimens and the vertical direction is in the range of 15–30°. There are usually two or three main cracks. However, the specimens of concrete under single and double axis compression have a higher degree of fragmentation, and the final failure is composed of multiple longitudinal cracks, and the integrity after failure is also poor [15], which is greatly different from the failure mode under ECC single and double axis.

At the same time, we observed the failure process of the specimen during the test, and found that during the entire compression failure process of all specimens, within the first 80% of the failure strength of the specimen, there was no significant change on the surface of the specimen, for specimens with low strain rates (strain rate of 10^−4^ s^−1^). When the specimen is close to the failure strength, a clear sound of PVA fiber breaking during the failure of the specimen can be heard, and it continues until the end of the experiment. For a specimen with higher strain rates (strain rates of 10^−3^, 10^−2^ s^−1^), when the specimen is close to the peak stress, the sound of dense fiber fracture inside the specimen can also be heard, and the frequency of fracture is far higher than lower strain rates. Moreover, the specimens maintain good integrity after they are damaged. Until the final loading stage of the test, there will be no material fragmentation due to the failure of the specimen. Due to the existence of the PVA fibers, the cracking of the specimen is limited, and a large amount of energy is absorbed near the peak stress. The PVA fiber inside the ECC has a good connection effect and improves the ductility and load-carrying capacity of the specimen. At the same time, due to the crack resistance and toughening effect of the PVA fiber, the specimens maintained good integrity, and they were only damaged along 2–3 main cracks. No explosion or fragmentation of concrete specimens happened.

### 3.2. Peak Stress

#### 3.2.1. ECC Biaxial Failure Criterion

The magnitude of the peak stress (ultimate compressive strength) depends on the response of the specimen under the ultimate load. See Table 4 for the peak stress under different side pressure levels. It can be seen from the table that the maximum peak stress is obtained when the lateral pressure level is 0.7.

**Figure 4:**
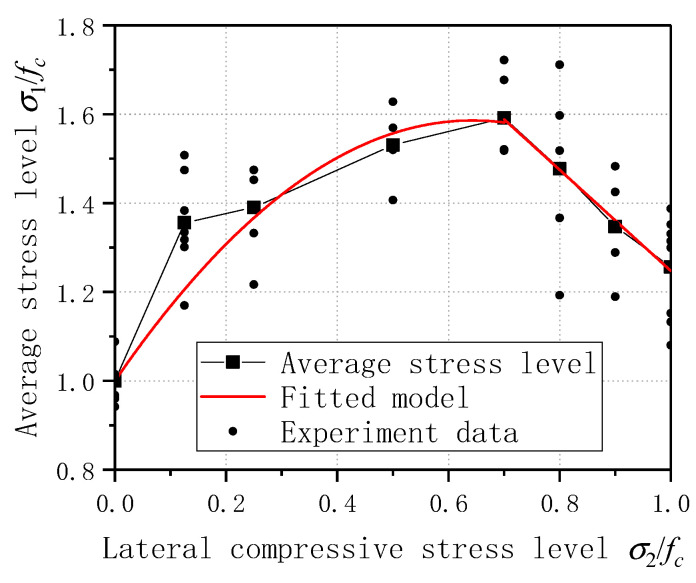
Fitting curve under ECC biaxial compression and experimental results under normalization.

As shown in Figure 4, the abscissa and ordinate axis is normalized with the uniaxial average compressive strength. Under the action of biaxial compressive stress, the principal compressive strength of ECC is significantly improved compared with the uniaxial compressive ultimate strength, which shows that the restraint provided by the side pressure improves the compressive strength of the sample. The increase of the main compression strength under biaxial is related to the stress ratio, and the strength of the main shaft is increased differently under different lateral compression strengths. When the lateral pressure is 0.125, 0.25, 0.5, 0.7, 0.8, 0.9, 1.0 *f_c_*, the average peak stress of the specimen is increased by 1.36, 1.39, 1.53, 1.59, 1.48, 1.35, and 1.46 times, respectively. The peak stress of ECC is the highest when the lateral pressure level C = 0.7, reaching 50.44 MPa. When the lateral pressure level is 0.125, the peak stress has been increased by 1.36 times, indicating that when a small amount of lateral restraint is provided, the compressive strength is significantly improved compared with uniaxial compression.

By observing the distribution of the sample’s average ultimate strength, when the lateral pressure level is 0–0.7, the part averages approximately a binomial distribution, and when the lateral pressure level is 0.7–1.0, it is roughly a linear distribution. On this basis, the ECC biaxial compression failure criterion is established:

(1) $$\frac{\sigma_{1}}{f_{c}}=1+1.822\frac{\sigma_{2}}{f_{c}}-1.4156{\left(\frac{\sigma_{2}}{f_{c}}\right)}^{2}(0\leq \frac{\sigma_{2}}{f_{c}}\leq 0.7)\;(a)$$

(2) $$\frac{\sigma_{1}}{f_{c}}=2.384-1.137\frac{\sigma_{2}}{f_{c}}\;(0.7\leq \frac{\sigma_{2}}{f_{c}}\leq 1)\;(b)$$

In Equation (1), *σ*_1_ represents the specimen’s longitudinal peak stress; *σ*_2_ is the lateral stress level of the specimen; fc is the average peak stress of the specimen. Among them, the correlation coefficient R^2^ of Equation (1) is 0.985, and the R^2^ of Equation (2) is 0.993. The fitting result is good and can be used to predict ECC’s compressive strength under different lateral pressure levels. The fitting curve corresponding to ECC biaxial compression failure criterion is given in Figure 4. The ECC biaxial compression failure criterion proposed in this paper can better predict the peak stress of ECC under biaxial compression stress.

Due to the random distribution of the fibers inside the ECC, the fibers inside the ECC have a bridging effect during the compression process, which greatly limits internal cracks. The failure of the specimen is in the form of oblique shear failure. Under biaxial compression, the lateral stress and fiber bridging jointly limit the development of cracks inside the ECC, and, thus, the strength is significantly improved. The stress ratio *σ*_2_/*f_c_* in the two directions determines the degree of increase in the ECC sample pressure and the size of the oblique shear pressure that the ECC can withstand. Therefore, the peak stress changes with the change of lateral pressure level C.

#### 3.2.2. Peak Stress under Different Lateral Pressure and Different Loading Rate

Table 5 shows the change of ECC peak stress under different lateral pressures and different strain rates. It can be seen from the table that under a certain lateral pressure, the ultimate strength of ECC increases with the increase of strain rate. At the same time, at a specific strain rate, with the rise of lateral pressure, the ultimate strength of ECC is increasing.

**Figure 5:**
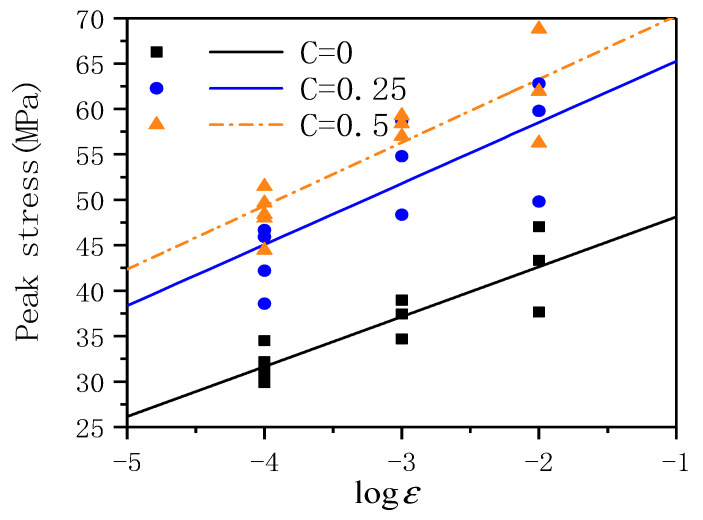
Relationship between peak stress and strain rate under different lateral pressure levels.

Figure 5 shows the relationship between peak stress and strain rate under different lateral pressure levels. With the increase of strain rate, the dynamic peak stress shows a linear increase trend, showing significant strain rate sensitivity. When the strain rate increases from 10^−4^ to 10^−2^ s^−1^, the average dynamic peak strength of the lateral pressure levels of C = 0, 0.25, and 0.5 increases by 37.5, 61.1, and 71.8%, respectively. To quantitatively evaluate the influence of the lateral pressure level and strain rate on the peak stress, the relationship between the peak stress and the strain rate under different lateral pressure levels is obtained by the fitting. The fitting curve is shown in the following formula:

(3) $$\begin{array}{ll} C=0 & \sigma_{\max}=53.61503+5.49528\dot{\varepsilon }\\ C=0.25 & \sigma_{\max}=71.99333+6.73034\dot{\varepsilon }\\ C=0.5 & \sigma_{\max}=77.23331+6.97402\dot{\varepsilon } \end{array}$$

In Equation (3), *σ*_max_ is the peak stress under the dynamic strain rate, $\dot{\varepsilon }$ is the dynamic strain rate. The correlation coefficient R^2^ is 0.999, 0.858, 0.895, respectively. The correlation coefficient R^2^ is relatively high, so it can be considered that the fitting curve can describe the relationship between the average compressive strength under different lateral pressures. The fitting curve can be found through that as the lateral pressure level increases, the intercept of the fitting curve increases, but the rate of increase decreases; as the lateral pressure level increases, the slope of the fitting curve gradually increases. When the strain rate keeps constant, with the lateral pressure growing, ECC’s average peak stress also keeps growing.

#### 3.2.3. Dynamic Intensity Growth Factor *DIF* − *σ*

For evaluating the strain rate sensitivity of a material’s strength, the dynamic strength growth factor *DIF* − *σ* value is generally used for research [21]. The calculation method of *DIF* − *σ* is:

(4) $$DIF-\sigma =\frac{\sigma_{1}}{\sigma_{0}}$$

Among them, *σ*_1_ represents the dynamic compressive strength under different lateral pressures and strain rates, and *σ*_0_ represents the static compressive strength of different lateral pressure levels (C = 0, 0.25, 0.5). In such conditions, the uniaxial crushing strength under the shape of a strain rate of 10^−4^ s^−1^ has functioned as the benchmark static crushing strength, and the calculated results are shown in Table 6.

**Figure 6:**
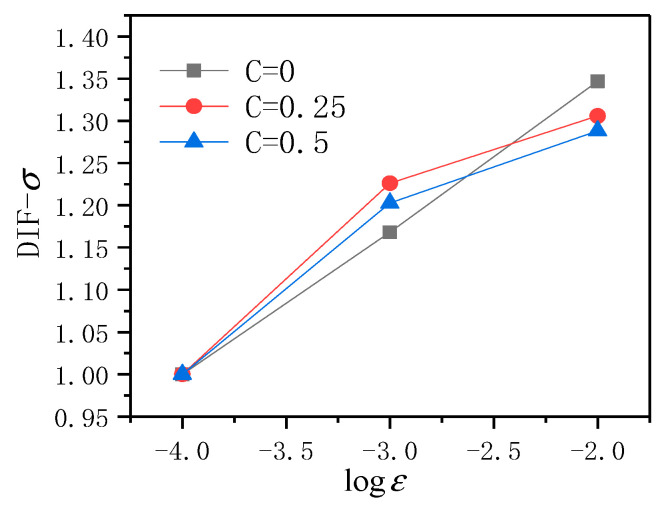
The broken line graph of *DIF* − *σ* of ECC with dynamic strain rate under different lateral pressure levels.

Draw all obtained data results into a more intuitive discount chart for analysis, as shown in Figure 6.

It can be found from Figure 6 that the strain rate sensitivity of ECC is different under different lateral pressure levels. In general, no matter how much the lateral pressure level, with the dynamic strain rate, grows, the average compressive strength of ECC has been improved. When the lateral pressure level C = 0, the *DIF* − *σ* value of ECC increases the most with the rise of the dynamic strain rate, followed by the lateral pressure level of 0.25, and when the lateral pressure level C = 0.5, the dynamic strain rate increases the least. Due to the existence of PVA fibers, the formation and development of cracks require high energy. The higher the loading rate is, the shorter the loading time is. From the energy point of view, insufficient time is given to the material to accumulate the corresponding energy through the generation and development or deformation of cracks, according to the energy principle, the material can only achieve energy balance by increasing the stress, so the average peak stress of ECC increases with the increase of the strain rate [21].

### 3.3. Peak Strain

Table 7 shows the peak strain of ECC under different lateral pressures and different stress levels. It can be seen from the table that when the lateral pressure level is constant, the peak strain in the vertical direction of ECC increases with the increase of the strain rate. When the strain rate is stable, the peak strain also increases with the lateral pressure increase. In Raymond’s biaxial plate HPFRC under compression, a similar conclusion was obtained [16].

**Figure 7:**
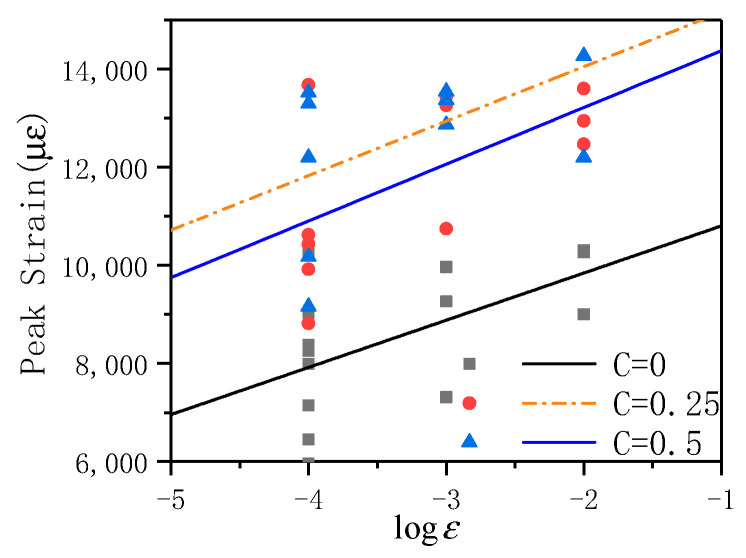
The relationship between peak strain and strain rate under different lateral pressure levels.

Figure 7 shows the relationship between peak strain and strain rate under different lateral pressure levels. When the strain rate grows from 10^−4^ to 10^−2^ s^−1^, the average peak of the lateral pressure levels of C = 0, 0.25, and 0.5 grows by 24.2, 63.8, and 74.9%, respectively. In order to quantitatively evaluate the influence of the lateral pressure level and strain rate on the peak strain, the relationship between the peak stress and the strain rate under different lateral pressure levels is obtained by fitting, and the fitting curve is shown in Equation (5):

(5) $$\begin{array}{ll} C=0 & \varepsilon_{\max}=11764.16+961.04\dot{\varepsilon }\\ C=0.25 & \varepsilon_{\max}=15527.15+1155.45\dot{\varepsilon }\\ C=0.5 & \varepsilon_{\max}=16268.45+1109.68\dot{\varepsilon } \end{array}$$

In Equation (5), $\varepsilon_{\max}$ is the ultimate peak strain under the dynamic strain rate, $\dot{\varepsilon }$ is the dynamic strain rate. The correlation coefficient R^2^ is 0.998, 0.818, 0.883, respectively. The correlation coefficients R^2^ are all high, and it can be considered that the fitted curve can describe the relationship between the average peak strains under different lateral pressures. The fitting curve can be found through that as the lateral pressure level increases, the intercept of the fitting curve increases, but the rate of increase decreases; with the lateral pressure level increases, the slope of the fitting curve does not change much. At the same time, when the strain rate is constant, with the increase of lateral pressure, the average ultimate peak strain of ECC is also increasing. Regarding the increase of ECC peak stress, Yin believes that the effect of adding fibers to concrete is equivalent to providing some small constraints on the free surface [25]. At the same time, due to the introduction of lateral pressure, under the combined action of these two factors, in the process of fiber being pulled out and broken, it is equivalent to the formation of a triaxial compression environment, so that the peak stress of ECC has been significantly improved.

(1) Dynamic strain growth factor *DIF* − *ε*

For evaluating the strain rate sensitivity of a material’s strain, the dynamic strain growth factor *DIF* − *ε* value is generally used for research [21]. The calculation method of *DIF* − *ε* is:

(6) $$DIF-\varepsilon =\frac{\varepsilon_{1}}{\varepsilon_{0}}$$

In Equation (6), *ε*_1_ signifies the dynamic peak strain at different lateral pressures and strain rates, and *ε*_0_ represents the static peak strain at different lateral pressure levels (C = 0, 0.25, 0.5). The uniaxial peak strain under a strain rate of 10^−4^ s^−1^ is taken as the reference dynamic peak strain. The calculated results are shown in Table 8.

**Figure 8:**
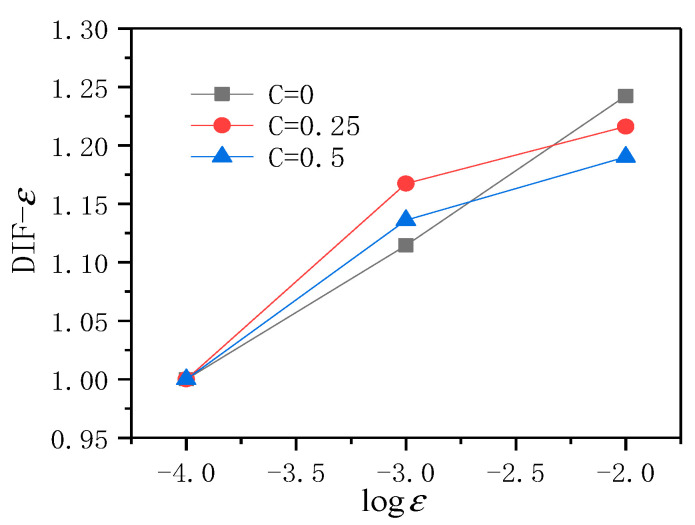
The broken line graph of *DIF* − *ε* of ECC with dynamic strain rate under different lateral pressure levels.

Draw all obtained data results into a more intuitive discount chart for analysis, as shown in Figure 8.

It can be seen from Figure 8 that, overall, no matter what the level of lateral pressure is, the *DIF* − *ε* of ECC will increase with the increase of strain rate. The rate of increase in *DIF* − *ε* slows down.

### 3.4. Deformation Modulus

Unlike linear elastic materials, the stress-strain relationship of ECC under compression is a curve. In different stress stages, the ratio of stress to strain is a variable, which cannot be called the elastic modulus, but deformation modulus. In general, there are three ways to express the deformation modulus of ECC:

(1) ECC elastic modulus (origin modulus) $E_{c}$. The slope of the tangent line at the origin of the ECC compressive stress-strain curve is the initial elastic modulus of the ECC.
(2) The deformation modulus of ECC (secant modulus) ${E_{c}}^{\prime }$, that is, ratio of peak stress to peak strain which is called the mean secant modulus of ECC.
(3) The tangent modulus of ECC ${E_{c}}^{\prime \prime }$ is the tangent slope at a specific stress value on the ECC stress-strain curve taken as the tangent modulus of ECC.

In order to comprehensively compare the deformation modulus of ECC under different lateral pressure and different strain rates, this paper takes the mean secant modulus ${E_{c}}^{\prime }$ (2) of ECC to evaluate the deformation modulus of ECC. Table 9 shows the deformation modulus of ECC under different strain rates and other lateral pressures. It can be seen from the table that under the same lateral pressure level, the elastic modulus of ECC increases, and under the same strain rate, the elastic modulus of ECC also increases.

**Figure 9:**
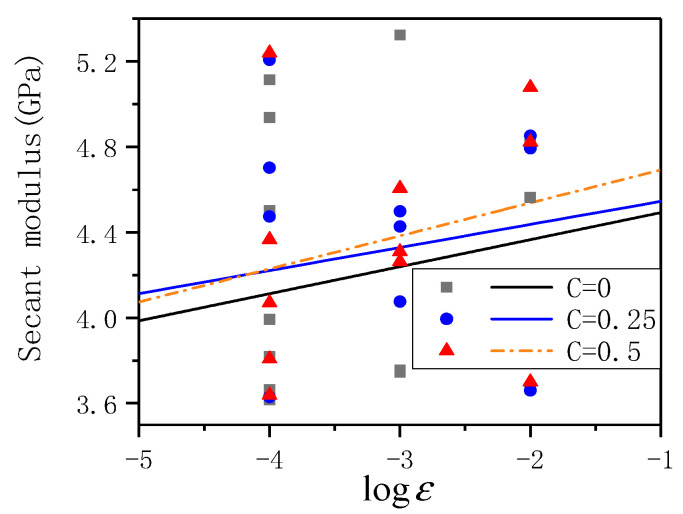
The relationship between deformation modulus and strain rate under different lateral pressure levels.

Figure 9 shows the variation of deformation modulus with strain rate under different lateral pressure levels. It can be seen from the figure that with the strain rate increases, the average deformation modulus of ECC increases to a certain extent. Still, the degree of improvement is limited, showing a specific strain rate sensitivity, but the deformation modulus of the specimen has a higher dispersion. Simultaneously, with the level of lateral pressure increases, the average deformation modulus of ECC also increases, but the increase is even smaller. To quantitatively evaluate the influence of lateral pressure level and strain rate on deformation modulus, the fitting curve of ECC average deformation modulus and strain rate is obtained through fitting:

(7) $$\begin{array}{ll} C=0 & {E_{c}}^{\prime }=4.61935+0.12654\dot{\varepsilon }\\ C=0.25 & {E_{c}}^{\prime }=4.65377+0.10799\dot{\varepsilon }\\ C=0.5 & {E_{c}}^{\prime }=4.84748+0.15453\dot{\varepsilon } \end{array}$$

In Equation (7) ${E_{c}}^{\prime }$ is the deformation modulus (GPa) under dynamic strain rate, $\dot{\varepsilon }$ is the dynamic strain rate. The correlation coefficient R^2^ is 0.890, 0.998, 0.995, respectively. R^2^ are all high; it can be considered that the fitted curve can describe the relationship between the average peak strain and under different lateral pressures. The fitting curve can be found that as the level of lateral pressure increases, the intercept of the fitting curve increases, but the slope first decreases and then increases.

### 3.5. Stress-Strain Curve

(1) ECC compressive stress-strain curve

**Figure 10:**
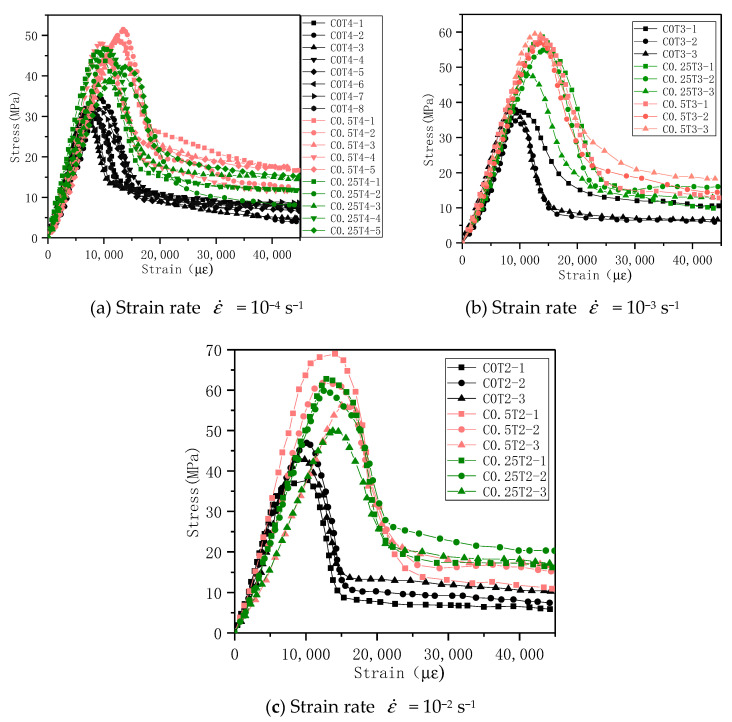
Compressive stress-strain curves under different lateral pressure levels.

According to the load value measured by the load sensor on the structural testing machine and the displacement change collected by the motion detector, the stress and average strain (displacement change/specimen height) of the specimen in each corresponding state can be calculated, and stress-strain curves were drawn. The stress-strain curves of different lateral pressure levels and different strain rates are shown in Figure 10. According to the shape of the stress-strain curve and the development of cracks, the entire compression process of ECC can be divided into the following four stages.

The first stage: the elastic stage, from the coordinate origin to the proportional limit point. The proportional limit is about 75% of the peak stress. At this stage, the matrix is elastically deformed under the action of an external load, the stress-strain curve is approximately linear, the matrix and the fiber bear the external load, and the matrix has no visible cracks. Occasionally, a crackle of fiber breaking inside the specimen can be heard during the loading process.

The second stage: the crack propagation stage; this stage is from the limit point of the proportion to the peak point. At this stage, when the external load is gradually increasing, the sound of continuous fiber rupture inside the specimen can be heard, and apparent cracks can be seen on the surface of the specimen. The stress-strain curve shows a curve growth and gradually reaches the peak stress.

The third stage: instability failure stage, from the peak point to the descending recurve point. In this stage, the specimens’ failure cracks have been completely formed, and the specimens are rapidly destroyed. The load decreases quickly with the intensification of deformation. At this stage, since most of the internal primary crack fibers have broken, in consequence, the load drops rapidly, but there will be no burst or collapse when compressing brittle materials such as concrete.

The fourth stage: residual stage. This stage is after the point of reverse bending. At this stage, due to the formation of the main crack of the specimen, the test piece having been damaged, so it is incapable of remaining an entirety, but a “mechanism” formed by partial fiber connections. At this stage, the sound of fiber breaking can still be heard, and the fibers are continuously pulled. When the load is pulled out or broken, the load slowly decreases under uniaxial, and the load remains stable and gradually decreases with the increase of strain under biaxial. Sometimes there is a small increase in stress. At this time, the specimen can still maintain peak stress of 20–30%.

**Figure 11:**
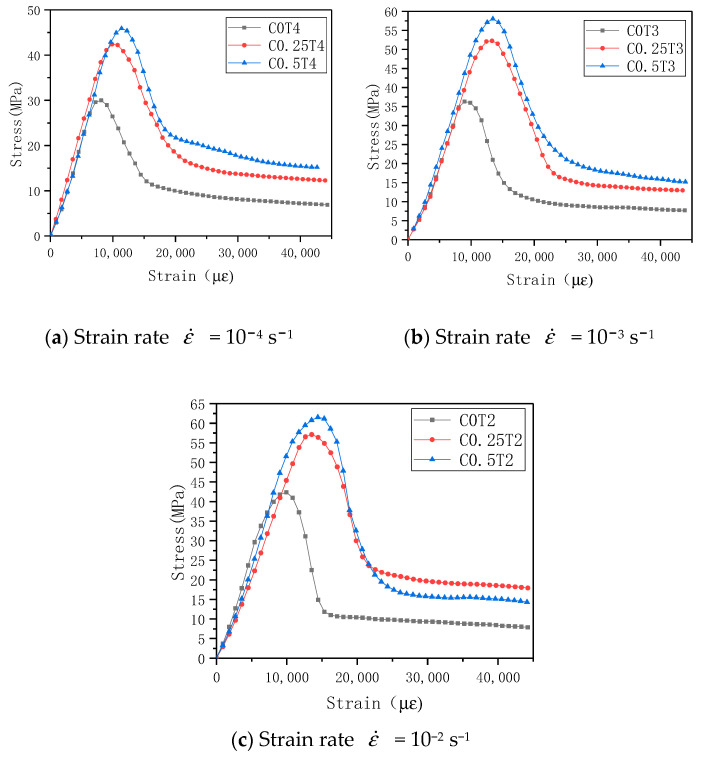
Average stress-strain curves at different lateral pressure levels corresponding to different strain rates.

The stress-strain curve pattern of each group of specimens under different lateral pressure levels is similar. Under the lateral pressure environment, the second half of the specimen stress-strain curve remains stable or continues to increase, but the increment is small. It can be understood as a pure plastic deformation section. From the stress-strain curves of the same group of specimens, it can be seen that the curves have a certain degree of dispersion. However, there is also a clear trend. To evaluate the influence of lateral pressure level and strain rate on the stress-strain curve, the stress-strain curve can be averaged, and the result can reflect the influence of the research variable on the stress-strain curve [26]. The average stress-strain curve of the specimen can be obtained by averaging the multiple stress-strain curves obtained in the same group of experiments, as shown in Figure 11. It can be found that the stress-strain curves of each group of specimens after averaging are similar in shape, and they are basically in line with the four-stage development law of stress-strain curves. When the strain rate is constant, with the increase of the lateral pressure level, the curve’s ultimate stress and the corresponding peak strain increase. ECC’s failure process is affected by the three effects of lateral restraint, strain hardening, and damage softening. Due to the fiber’s action, after the curve passes the peak stress point, the load gradually decreases with the increase of deformation, and there will be no momentary drop in the load of the concrete material that suddenly fails.

(2) Constitutive relationship

**Figure 12:**
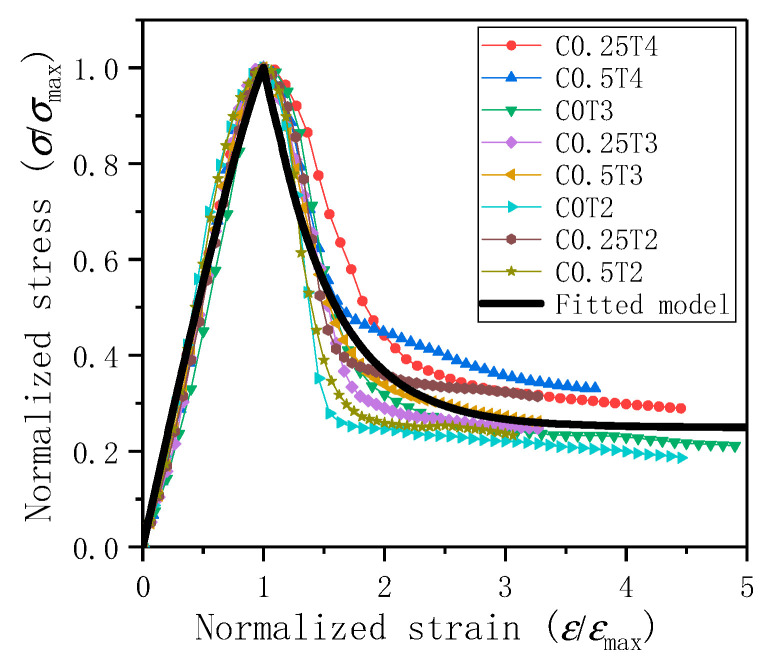
ECC stress-strain relationship under normalization and the normalized constitutive relationship established.

The ECC compressive stress-strain curves at different lateral pressure levels and strain rates are normalized based on the peak stress and peak strain. The normalized curve is shown in Figure 12. The shape of the curve under different lateral pressure and different strain rates is similar and can be described by the same set of fittings. According to the curve form, a two-stage formula is selected for fitting, see Equation (8).

(8) $$\frac{\sigma }{\sigma_{\max}}=\{\begin{array}{ll} 1-{\left(1-\frac{\varepsilon }{\varepsilon_{\max}}\right)}^{A_{0}} & \frac{\varepsilon }{\varepsilon_{\max}}\leq 1\\ A_{1}\exp\left(-\frac{\varepsilon }{\varepsilon_{\max}A_{2}}\right)+A_{3} & \frac{\varepsilon }{\varepsilon_{\max}}>1 \end{array}$$

In the Equation (8): *A*_0_ = 1.16312, correlation coefficient R^2^ = 0.98; *A*_1_ = 5.25844, *A*_2_ = 0.52526, *A*_3_ = 0.24944; correlation coefficient R^2^ = 0.94. The fitting degree is relatively high and can be used to describe the constitutive relationship of ECC under different lateral pressures and strain rates. Suppose the peak stress and peak strain of ECC are known, on the basis of obtaining a set of static compressive peak stress and peak strain. In that case, ECC’s dynamic compression constitutive relationship under different lateral pressure and different strain rates can be roughly fitted.

(3) Compressive toughness index

**Figure 13:**
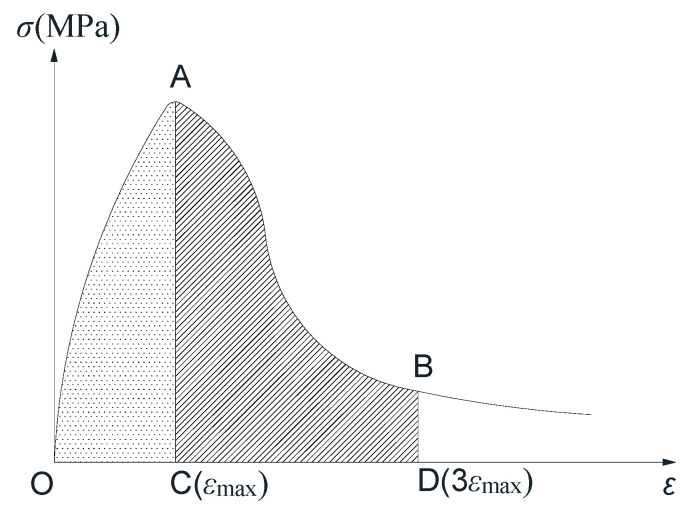
ECC typical full curve of compressive stress and strain.

The degree of compressive stress softening, or the second half of the stress-strain curve after the peak stress depends on the ECC’s cracking and the full use of the connection between the fiber and the matrix due to the cracking. To quantitatively compare the influence of lateral pressure level and strain rate on the dynamic compressive toughness of ECC specimens, it is necessary to establish a reasonable evaluation index of compressive toughness [26]. The easiest way to quantify compressive toughness is to use the toughness index [21]. When the compressive strain reaches three times the peak strain ε_max_, the curve after the peak can fully reflect the material’s compressive toughness. Therefore, in this study, the toughness index is defined as the stress-strain curve area’s ratio to the area enclosed by the *x*-axis and the peak strain ε_max_ when the strain value reaches three times the peak strain, as is shown in Figure 13.

In Equation (9), CTI represents the compressive toughness index, S_OABD_ represents the area of the shadow surrounded by O, A, B, and D in Figure 13, and S_OAC_ represents the shadow surrounded by O, A, and C The size of the part of the area.

(9) $$\mathrm{CTI}=\frac{S_{\mathrm{OABD}}}{S_{\mathrm{OAC}}}$$

**Figure 14:**
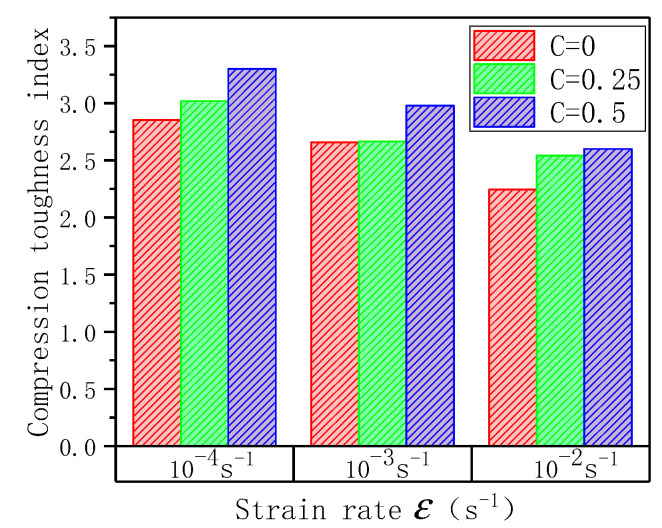
Compressive toughness index of specimens under different lateral pressure levels at different strain rates.

After calculation, ECC’s toughness index at different lateral pressure levels and strain rates is obtained, as shown in Figure 14. It can be found from the figure that, overall, the compressive toughness index of ECC is 2.25–3.30. When the strain rate is constant, as the lateral pressure level increases, the compressive toughness index of ECC is continuously increasing, from 10^−4^ to 10^−2^ s^−1^, the compressive toughness indexes were 3.30, 2.98, and 2.60, which increased by 15.66, 12.05, and 15.82%, respectively, compared to when there was no lateral pressure to the lateral pressure level C = 0.5. This is because of lateral restraint, which restrains the specimen’s deformation after cracking, and the cracks of ECC can fully develop and give full play to the bridging effect of the PVA fiber. At the same time, it can be found that when the strain rate is constant, with the increase of the strain rate, the compressive toughness index of ECC is continuously decreasing. When C = 0.5, the compressive toughness index is 2.25, 2.54, 2.60, respectively, compared with C = 0; the compressive toughness index declined by 27.10, 18.81, and 26.93% respectively. This is because the increase in loading rate harms the connection between the PVA fiber and the matrix. Through numerical simulation, it is found that the loading rate has a significant effect on the failure of the drawing specimen. At high strain rates, the failure unit of the specimen matrix increases significantly [27]. With the strain rate growing, the ratio of fiber fracture inside the specimen and pullout may have changed to some extent. When the lateral pressure level is constant, with the increase of strain rate, ECC tends to be brittle. Since the bridging of fibers inside the ECC can still play an effective role, the degree of compressive toughness index decline is not large.

### 3.6. Comparison with Other Researchers

**Figure 15:**
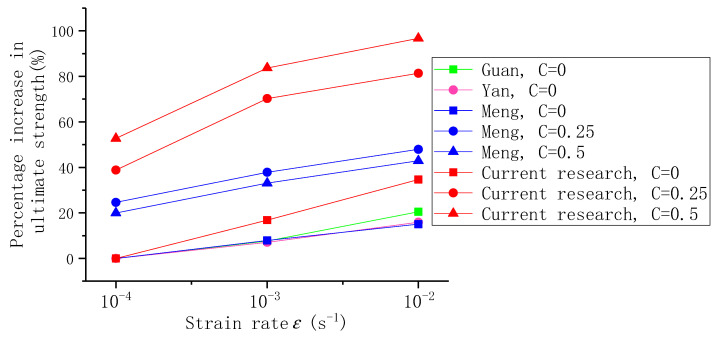
Increasing percentage of ultimate strength with ratio (%).

To better compare the results of this paper with those of other researchers under biaxial dynamics, we compared the results of other researchers. The results of the comparison can be found in Table 10. For a clearer comparison, the results in Table 10 are plotted in Figure 15. As can be seen from Figure 15, the percentage increase in ECC limit strength is significantly greater than that of plain concrete as the strain rate increases. At the same time, when the strain rate is certain, at the lateral pressure level of 0.25, the ECC limit strength is significantly higher than the concrete. However, a significant difference can be observed. At a certain rate of strain, as the lateral pressure level increases, the limit strength of the ECC increases, with the maximum limit strength at C = 0.5. For plain concrete, with the increase of lateral pressure level, the strength of concrete is not always increasing, reaching the highest level at C = 0.25. By comparison, it can be found that the performance of ECC in the ultimate strength increase under the biaxial dynamic is significantly better than that of plain concrete. In future research, we can study whether the dynamic performance of ECC structure is significantly better than that of concrete structure, and provide reference for the high strain rate environment of future ECC material service.

## 4. Conclusions

In this paper, an improved hydraulic servo structure testing machine has been used to conduct biaxial dynamic compression tests on eight types of engineered cementitious composites (ECC) with lateral pressure levels of 0, 0.125, 0.25, 0.5, 0.7, 0.8, 0.9, and 1.0, and three strain rates of 10^−4^, 10^−3^, and 10^−2^ s^−1^. From the test results and analysis, the following conclusions are drawn:

(1) Under uniaxial compression, due to the internal bridging effect of the ECC specimen, the failure form of the specimen is a shear failure, and the main cracks of the specimen are generated on both surfaces. Under various lateral pressure stress levels, the failure form of the specimen is also shear failure, but the main crack direction appears on the lateral pressure surface. The significant cracks are generally 2–3, and the cracking direction is 15~30° with the longitudinal direction. The strain rate has little effect on the final failure form of the specimen.
(2) Under the condition of biaxial compressive stress, ECC’s peak stress is higher than that under uniaxial. When the lateral pressure level is 0.7, the specimen’s peak stress reaches the highest, which is increased by 59%. Based on the normalized stress level, the failure criterion of ECC under biaxial is established, and the fitting effect is good, which can be used to predict the peak stress of ECC under different lateral pressures. Through dynamic strength growth factor *DIF* − *σ* and dynamic strain growth factor *DIF* − *ε*, the effect of strain rate on ECC peak stress and strain under different lateral pressure levels was evaluated. With the lateral pressure increases, the peak stress of ECC increases continuously. At the same time, under a certain level of lateral pressure, with the strain rate increases, the peak strain of ECC is also growing.
(3) Under different lateral pressure levels and other stress levels, the deformation modulus of ECC has greater dispersion, but overall, the increase in lateral pressure and strain rate increases the average deformation modulus of ECC; however, the growth of deformation modulus is small. Through fitting, it is found that the ECC deformation modulus and the strain rate increase approximately linearly with the growth of the lateral pressure level.
(4) The stress-strain curve of ECC under different lateral pressures and different strain rates can be divided into four stages: elastic stage, crack propagation stage, crack instability propagation stage, and residual stage. The compressive toughness index quantitatively evaluated the compressive toughness of ECC. When the strain rate is constant, as the lateral pressure level increases, the compressive toughness of ECC increases; when the lateral pressure level is constant, as the strain rate increases, ECC’s compressive toughness gradually decreases.
(5) A normalized ECC constitutive relationship is established. The known static compression test results can be used to estimate the full curve of ECC compressive stress and strain under different lateral pressure levels and different strain rates.
(6) Compared with concrete, the ultimate strength improvement of ECC under biaxial dynamic conditions is significantly higher than that of plain concrete. In the future, we can explore the application of ECC structures in high strain rate and complex stress environments.

## Figures and Tables

**Table 1:** Polyvinyl alcohol (PVA) fiber parameters.

Diameter (mm)	Length (mm)	Length Diameter Ratio	Elongation (%)	Tensile Strength (MPa)	Young’s Modulus (GPa)	Density (kg/m^3^)
0.04	12	300	6	1600	40	1300

**Table 2:** Mix proportion.

Number	Cement	Fly Ash	Silicon Sand	Silica Dume	Water	Reducer	PVA Volume Content
M8	1	3.9	1.25	0.1	1.25	0.725%	2%

Note: The volume content is used for PVA fiber, and the weight ratio is used for other constituent materials.

**Table 3:** Loading conditions.

Sample Serial Number	Strain Rate (s^−1^)	Lateral Pressure Level C	Amount
C0T4-x	10^−4^	0	8
C0.125T4-x		0.125	8
C0.25T4-x		0.25	5
C0.5T4-x		0.5	5
C0.7T4-x		0.7	5
C0.8T4-x		0.8	5
C0.9T4-x		0.9	5
C1.0T4-x		1	8
C0T3-x	10^−3^	0	3
C0.25T3-x		0.25	3
C0.5T3-x		0.5	3
C0T2-x	10^−2^	0	3
C0.25T2-x		0.25	3
C0.5T2-x		0.5	3

Note: In the sample number, C represents the lateral pressure, the number after C represents the lateral pressure level; the number after T and T represents different loading rates, and x represents the sample number of the same group of samples.

**Table 4:** ECC ultimate strength test results under different lateral pressures (MPa).

Sample Serial Number	Lateral Pressure Level C							
	0	0.125	0.25	0.5	0.7	0.8	0.9	1
1	29.85	43.84	46.02	48.47	54.55	50.61	37.68	48.96
2	34.49	43.07	46.73	51.58	48.19	54.23	40.85	47.82
3	30.42	42.28	38.56	49.74	48.19	43.31	45.15	49.75
4	32.17	41.75	46.72	48.16	53.15	37.80	42.79	41.68
5	32.14	41.23	42.23	44.57	48.11	48.11	46.99	51.06
6	31.85	46.71						42.39
7	31.93	37.06						48.37
8	30.66	47.78						39.75
Average	31.69	42.97	44.05	48.51	50.44	46.81	42.69	39.81

**Table 5:** Peak stress (MPa) at different lateral pressures and different strain rates.

Lateral Pressure Level C	Strain Rate(s^−1^)						
	No.	10^−4^	No.	10^−4^	No.	10^−3^	10^−2^
0	1	29.87	5	32.16	1	37.42	37.66
	2	34.48	6	31.86	2	34.69	47.05
	3	30.46	7	31.92	3	38.94	43.33
	4	32.09	8	30.68			
Average		31.69				37.02	42.68
0.25	1	45.92	4	46.67	1	58.71	62.80
	2	46.66	5	42.18	2	54.79	59.78
	3	38.56			3	48.34	49.80
Average		44.00				53.95	57.46
0.5	1	48.38	4	48.00	1	58.36	68.81
	2	51.47	5	44.44	2	56.98	61.95
	3	49.66			3	59.26	56.26
Average		48.39				58.20	62.34

**Table 6:** Dynamic intensity growth factor (*DIF*) − *σ* values of ECC under different lateral pressure levels.

Lateral Pressure Level C	Strain Rate (s^−1^)	*DIF* − *σ*	Increasing Rate of *DIF* − *σ*
0	10^−4^	1.00	0%
	10^−3^	1.17	17%
	10^−2^	1.35	35%
0.25	10^−4^	1.00	0%
	10^−3^	1.23	23%
	10^−2^	1.31	31%
0.5	10^−4^	1.00	0%
	10^−3^	1.20	20%
	10^−2^	1.29	29%

**Table 7:** ECC peak strain under different lateral pressure and different strain rates (*με*).

Lateral Pressure Level C	Strain Rate (s^−1^)						
	No.	10^−4^	No.	10^−4^	No.	10^−3^	10^−2^
0	1	8259.43	5	7144.43	1	9964.45	10,267.59
	2	9029.85	6	6453.55	2	9262.64	10,308.43
	3	5955.65	7	7993.91	3	7313.08	9001.68
	4	10,286.86	8	8373.49			
Average		7937.14				8846.72	9859.23
0.25	1	8818.28	4	10,430.87	1	13,261.65	12,942.86
	2	9921.37	5	13,676.69	2	13,441.80	12,469.28
	3	10,623.42			3	10,746.32	10,746.32
Average		10,694.12				12,483.26	13,005.03
0.5	1	13,296.65	4	9160.04	1	13,543.13	14,267.89
	2	13,519.30	5	10,176.52	2	13,366.30	12,199.68
	3	12,199.19			3	12,865.01	15,201.56
Average		11,670.34				13,258.15	13,889.71

**Table 8:** *DIF* − *ε* values of ECC under different lateral pressure levels.

Lateral Pressure Level C	Strain Rate (s^−1^)	*DIF* − ε	Increasing Rate of *DIF* − ε
0	10^−4^	1.00	0%
	10^−3^	1.11	11%
	10^−2^	1.24	24%
0.25	10^−4^	1.00	0%
	10^−3^	1.17	17%
	10^−2^	1.22	22%
0.5	10^−4^	1.00	0%
	10^−3^	1.14	14%
	10^−2^	1.19	19%

**Table 9:** ECC deformation modulus under different strain rates and different lateral pressure levels (GPa).

Lateral Pressure Level C	Strain Rate (s^−1^)						
	No.	10^−4^	No.	10^−4^	No.	10^−3^	10^−2^
0	1	3.62	5	4.50	1	3.76	3.67
	2	3.82	6	4.94	2	3.75	4.56
	3	5.11	7	3.99	3	5.32	4.81
	4	3.12	8	3.66			
Average		4.10				4.28	4.35
0.25	1	5.21	4	4.47	1	4.43	4.85
	2	4.70	5	3.08	2	4.08	4.79
	3	3.63			3	4.50	3.66
Average		4.22				4.33	4.44
0.5	1	3.64	4	5.24	1	4.31	4.82
	2	3.81	5	4.37	2	4.26	5.08
	3	4.07			3	4.61	3.70
Average		4.22				4.39	4.53

**Table 10:** Research results of strength by other researchers under different lateral pressure and strain rates.

Researcher	Uniaxial Ultimate Strength (MPa)	Material	Lateral Pressure Level C	The Percentage Increase of Ultimate Strength under Different Strain Rates (%)		
				10^−4^ S^−1^	10^−3^ S^−1^	10^−2^ S^−1^
Guan [14]	20.1	Plain concrete	0	0.00	7.63	20.49
Yan [12]	9.84	Plain Concrete	0	0.00	7.05	15.89
Meng [15]	34.36	Plain Concrete	0	0.00	7.87	15.03
			0.25	24.64	37.82	47.91
			0.5	19.96	33.11	42.91
Current research	31.69	ECC	0	0.00	16.82	34.68
			0.25	38.85	70.24	81.32
			0.5	52.70	83.65	96.72

## Data Availability

The data presented in this study are available on request from the corresponding author. The data are not publicly available due to the further research.
